# On respiratory droplets and face masks

**DOI:** 10.1063/5.0015044

**Published:** 2020-06-01

**Authors:** Talib Dbouk, Dimitris Drikakis

**Affiliations:** University of Nicosia, Nicosia CY-2417, Cyprus

## Abstract

Face mask filters—textile, surgical, or respiratory—are widely used in an effort to limit the spread of airborne viral infections. Our understanding of the droplet dynamics around a face mask filter, including the droplet containment and leakage from and passing through the cover, is incomplete. We present a fluid dynamics study of the transmission of respiratory droplets through and around a face mask filter. By employing multiphase computational fluid dynamics in a fully coupled Eulerian–Lagrangian framework, we investigate the droplet dynamics induced by a mild coughing incident and examine the fluid dynamics phenomena affecting the mask efficiency. The model takes into account turbulent dispersion forces, droplet phase-change, evaporation, and breakup in addition to the droplet–droplet and droplet–air interactions. The model mimics real events by using data, which closely resemble cough experiments. The study shows that the criteria employed for assessing the face mask performance must be modified to take into account the penetration dynamics of airborne droplet transmission, the fluid dynamics leakage around the filter, and reduction of efficiency during cough cycles. A new criterion for calculating more accurately the mask efficiency by taking into account the penetration dynamics is proposed. We show that the use of masks will reduce the airborne droplet transmission and will also protect the wearer from the droplets expelled from other subjects. However, many droplets still spread around and away from the cover, cumulatively, during cough cycles. Therefore, the use of a mask does not provide complete protection, and social distancing remains important during a pandemic. The implications of the reduced mask efficiency and respiratory droplet transmission away from the mask are even more critical for healthcare workers. The results of this study provide evidence of droplet transmission prevention by face masks, which can guide their use and further improvement.

## I. INTRODUCTION

Respiratory droplet transmission is considered critical for the rapid spread and continued circulation of viruses in humans.^1^ The droplets are produced by sneezing, coughing, or breathing, and the flu virus can exist even in the tiny droplets resulting from breath or speech alone.^2^ In a recent paper, Dbouk and Drikakis^3^ showed that human saliva-disease-carrier droplets could travel unexpected considerable distances depending on environmental conditions.

The SARS-CoV-2 pandemic has intensified the discussions about social distancing, the use of face masks, and other personal protective equipment (PPE). Therefore, the public and policymakers need to deepen their understanding of the degree of protection required and adjust to social distancing measures based on scientific evidence. Furthermore, we need to carefully assess the criteria used for evaluating the performance of face masks and PPEs and the multiphysics processes (e.g., fluid and particles dynamics) that can adversely impact their efficiency.

Hui *et al.*^4^ investigated the air dispersion distances traveled during the coughing of a human patient simulator using a laser visualization technique with smoke as a marker. They reported results with and without a surgical and an N95 mask. They showed that a normal cough induces a turbulent flow that spreads about 70 cm from the subject. The N95 mask prevented air leakage more effectively than the surgical mask during coughing, but there was still significant sideway leakage.

Using the Schlieren optical method, Tang *et al.*^5^ showed that wearing a standard surgical mask blocks the forward jet of droplets but allows leakage around the top, bottom, and sides. Furthermore, they showed that an N95 mask reduces the droplet leakage around the mask edges during the cough. However, the pressure inside the mask increases during coughs and the turbulent jet is directed through the front. Although both surgical and N95 masks decelerate the turbulent jet, none of them will prevent the droplets entirely from penetrating or escaping the mask, i.e., droplet transmission.

Mask efficiency is defined as the percentage of a contaminant removed by the mask filter. The mass, weight, number of particles, or volume can quantify it.^6^ Certification standards^7,8^ usually define a surgical mask efficiency as a constant value independent of coughing incidents or cycles. These standards neglect the fluid flow dynamics effect and droplet leakage through the mask openings. They also ignore the fact that the mask efficiency can deteriorate considerably over time due to saturation effects. Therefore, we need to take into account the cough and fluid dynamics in calculating the mask efficiency. Cyclic coughing incidents encompass complex fluid dynamics and vary across subjects. There is no objective system for assessing the frequency variation of coughs in patients. Furthermore, we do not know if the number of coughs is a function of the severity of the disease underlying the cough.^9,10^ Chronic coughers exhibit a significantly higher number of coughs throughout the day vs asthmatic patients.^9^ The above factors are essential in assessing the mask performance.

The main mechanisms of filtering through masks are droplets diffusion, interception, and impaction. During continued or cyclic coughing, the flow will increase and will adversely affect the mask filter efficiency. A wide range of filter efficiencies has been reported.^11–15^ The leakage of droplets out of the mask is also an essential factor that needs to be statistically quantified. Previous studies report limited effectiveness in using surgical masks to reduce respiratory illness,^16–18^ and clinical trials report little effect on infection rates with and without surgical masks.^19,20^ In contrast, laboratory studies concerning coughing and infectious subjects showed that surgical masks are effective at reducing the emission of large droplets^21,22^ and minimizing the lateral dispersion of droplets. However, they allowed simultaneous displacement of aerosol emission upward and downward from the mask.^4^ Several randomized trials have not found statistical differences in the effectiveness of surgical masks vs N95 filtering face-piece respirators (FFRs) at reducing respiratory diseases for healthcare workers.^23,24^

Given the above, this study aims to deepen our understanding of the fluid dynamics of respiratory droplets through and around face mask filters. We shed light on two important questions:

1. Do the complex fluid physics and cough dynamics result in altering the efficiency of face masks?
2. To what extent does the use of face masks reduce the distance of respiratory droplets transmission?

Scientific evidence to these questions will allow better use of personal protective equipment for both healthcare workers and the wider public. The results will also equip mask manufacturers and regulatory bodies with new knowledge. Finally, the results give insight into respiratory droplet transmission when wearing a mask, thus enabling a better appreciation of the protection offered by masks against airborne droplet carriers of viruses.

## II. MODELING

### A. Droplets initial size distribution

**FIG. 1.**
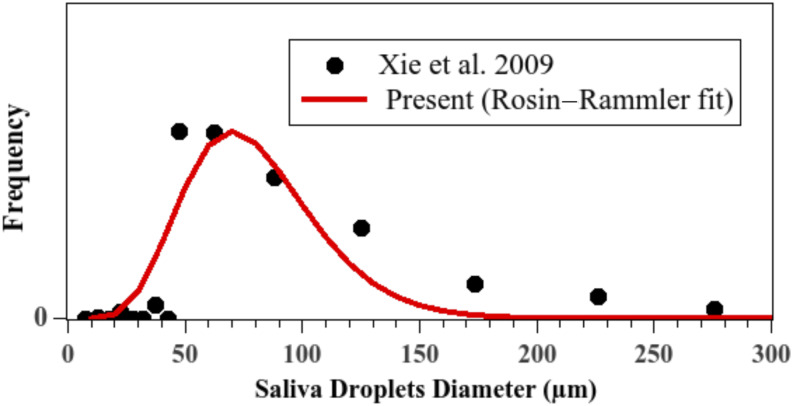
Initial saliva droplets size distribution. Reproduced with permission from T. Dbouk and D. Drikakis, “On coughing and airborne droplet transmission to humans,” Phys. Fluids **32**, 053310 (2020). Copyright 2020 AIP Publishing LLC.

The initial size distribution of droplets (Fig. 1) is the same as in the study of Dbouk and Drikakis,^3^ taken in the range of [1 *μ*m, 300 *μ*m] with 80 *µ*m as the mean diameter. This initial size distribution is very close to the data obtained by Xie *et al.*^25^ fitted using a Rosin–Rammler distribution law,^26^ also known as a Weibull distribution.^27^

### B. Initial and boundary conditions

**FIG. 2.**
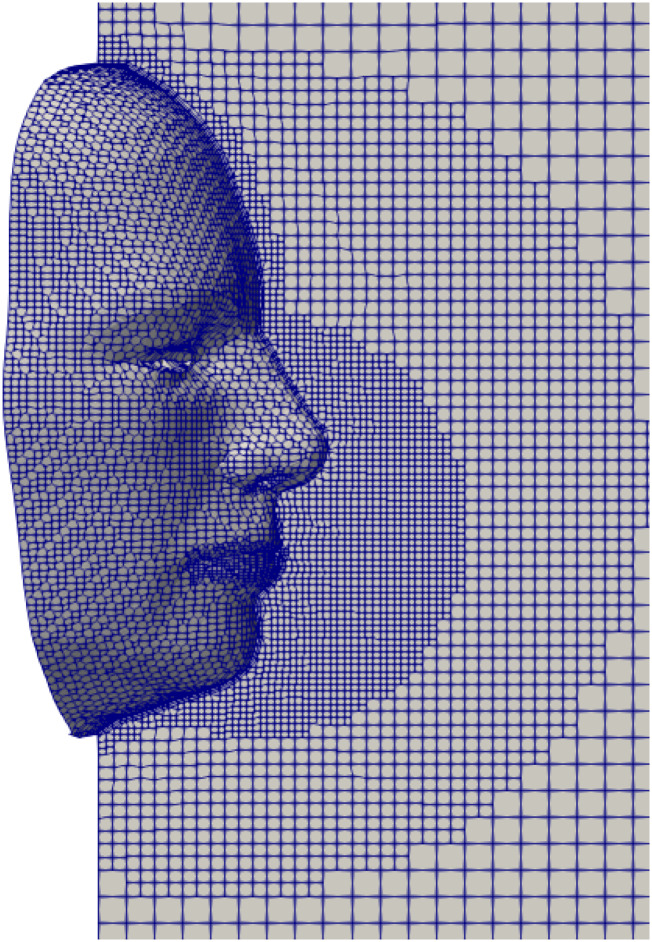
A 2D slice of the 3D computational domain using an advanced technique employing a hexahedral non-uniform structured mesh (≈0.52 × 10^6^ cells). The mesh is refined at the mouth-print, nose-print, and the face and then coarsened gradually in the streamwise cough flow direction with a multilevel refinement procedure. The overall computational domain dimensions are L = 1.6 m, W = 0.5 m, and H = 0.45 m.

The computational domain is three-dimensional, and the computational mesh comprises hexahedral non-uniform structured cells (≈0.52 × 10^6^), as shown in Fig. 2. The height between the top and bottom vertical planes is H = 0.45 m, the length of the domain is L = 1.6 m, and the width is W = 0.5 m. Figure 2 shows only a close-up part of the domain around the face. We refined the mesh near the mouth-print, the face, and the nose and then gradually coarsened it in the streamwise (cough flow) direction using a multi-level mesh technique. Using the grid convergence index (GCI) of Celik *et al.*,^28^ we conducted a mesh convergence study for the flow variables (fluid velocity, *u*_*f*_, and pressure, *p*) in a zone of interest in front of the mouth and nose enveloping the mask. Three grids were generated: 1-fine (1 015 154 cells), 2-medium (512 268 cells), and 3-coarse (350 132 cells) corresponding to refinement factors of *r*_21_ = 1.98 and *r*_32_ = 1.46, respectively. We adopted a medium-sized mesh, based on the results for the GCI (%): *GCI*_21_ = 3.5% and *GCI*_32_ = 4.5% for pressure and *GCI*_21_ = 3% and *GCI*_32_ = 4.45% for velocity.

**FIG. 3.**
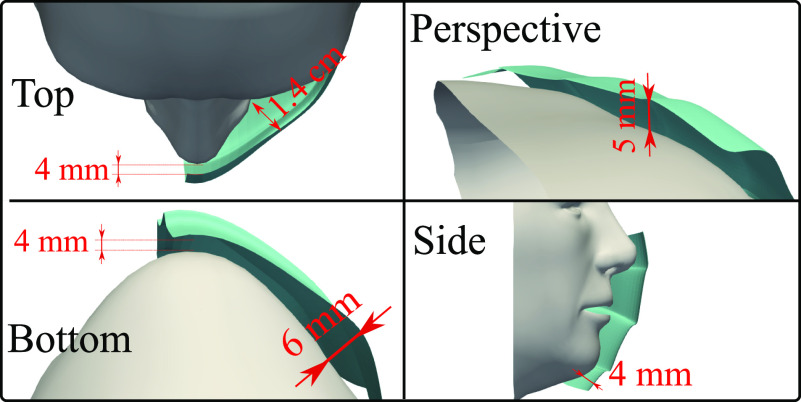
Face mask fitting.

We illustrate the mask fitting to the face in Fig. 3 showing different views (top, bottom, side, and perspective or rotated). The minimum distance between the mask and face is 4 mm, while the maximum distance is 1.4 cm located at the top view between the mask and the nose-to-eye corner. The above fitting scenario is close to reality. The appropriate situation will vary across subjects depending on many factors such as face morphology and its coherence to the curvature of the mask borders, amongst others. Our objective is to examine the fluid physics of airborne droplets that affects mask performance rather than performing a parametric study of all possible scenarios.

**FIG. 4.**
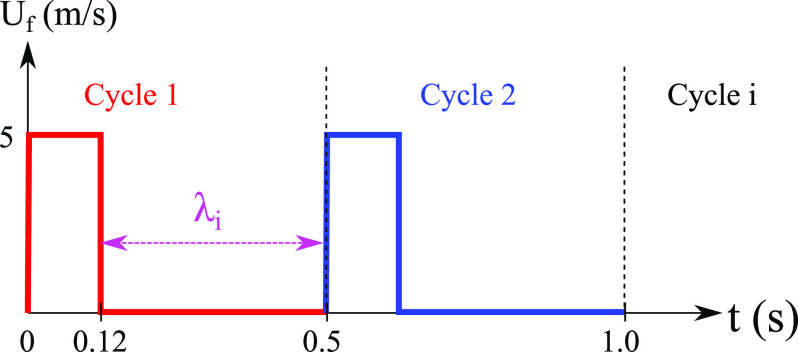
Cyclic conditions for the mouth inlet velocity *U*_*f*_ mimicking human coughing as observed experimentally by Hsu *et al.*^9^ *λ*_*i*_ = 0.38 s with *i* ∈ [1, 10].

The initial total mass of the injected saliva into the field is 7.7 mg, with 1008 droplets at the mouth. The above data are of the same order of magnitude as in the literature.^25,29^ To mimic a real coughing incident, we injected, in each cycle (Fig. 4), 0.15 mg of droplets from the nose. Any initial or boundary conditions that we do not detail here match implicitly those in the study of Dbouk and Drikakis.^3^ The wind speed is zero. The bottom plane boundary is an infinite domain.

In the study of Dbouk and Drikakis,^3^ we modeled a coughing incident by applying a time-varying velocity inlet with particle injected at the mouth boundary to mimic the human cough over 0.12 s. The objective was to examine the wind effect on airborne droplet transmission over a distance beyond 2 m (6 feet), and for this case study, the human cough over 0.12 s was sufficient.

In this paper, the velocity field is applied over ten cycles for a total period of 5 s (Fig. 4) such that the number of injected saliva droplets is repeated per cycle. We produced these ten cough cycles according to actual coughing incidents^9^ aiming to examine the droplet dynamics in detail. We have chosen the face skin temperature at 32 °C. The fluid properties and the Reynolds number are the same as those in the study of Dbouk and Drikakis.^3^ It is based on the mouth-print hydraulic diameter (*D*_*h*_) and its value is Re = 4400 (*Re* = *U*_*f*_*D*_*h*_/*ν*_*f*_), where *U*_*f*_ is the cough flow velocity inlet at the mouth and *ν*_*f*_ is the fluid kinematic viscosity (for air as an ideal gas). The nose fluid velocity inlet *U*_*n*_ was applied as *U*_*n*_ = *U*_*f*_/20 to mimic a real cough situation, where fewer droplets are expelled from the nose at a significantly lower speed.

### C. Fluid physics model

**FIG. 5.**
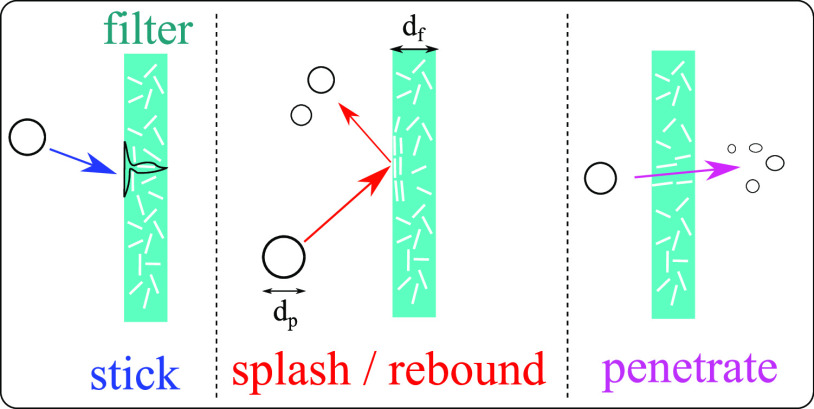
Droplet filter surface interaction modes.

We applied Eulerian–Lagrangian multiphase modeling with a full coupling between the phases. We also took into account the drag and gravitational forces, droplet breakup,^30^ droplet evaporation,^31,32^ and the turbulent dispersion forces.^33^ The governing equations are detailed in many textbooks.^34,35^ For the carrier bulk multiphase fluid mixture, we have employed the compressible Reynolds-averaged Navier–Stokes equations in conjunction with the *k*–*ω* turbulence model in the shear-stress-transport formulation.^36^ The modeling approach for the fluid (air–water vapor mixture) and the saliva droplet (liquid water) phases are detailed in the study of Dbouk and Drikakis.^3^ In this paper, we focus on modeling the transition laws (or modes) for a droplet impacting a porous wall of thickness *d*_*f*_ representing the thickness of the mask filter (Fig. 5). The alternative would be to use an empirical law for filter efficiency as a function of a droplet diameter and generate a random number of droplets penetrating the cover shield.^37^ However, we believe that taking into account the local interactions and implementing transition laws are essential components to the model for predicting accurately the overall efficiency of a face mask filter, as described in Sec. II D. The open-source computational fluid dynamics code “OpenFOAM”^38^ (version 7) within the framework of the finite volume method^39^ was employed. Furthermore, we carried out numerical code developments to extend the solver in OpenFOAM to account for local droplet interactions impacting a porous medium. The computations were performed on 32 intel-Xeon processors of 3 GHz. The computational time was ∼3 days and 1.5 days for the cases with and without a mask, respectively.

### D. Interaction modes

Past studies reported results for droplet dynamics such as wettability, the droplet size distribution, and penetration through gaps and filtering media across different applications.^40–46^ All the above studies and references therein are relevant to droplet penetration through filters, the droplet size distribution, and droplet diffusion, convection, Brownian motion, or Darcy flow. In this study, following the study of Bai *et al.*,^45,46^ droplet-impermeable-wall local interaction modes are described by the two dimensionless numbers: the droplet Weber number (We) and the droplet Laplace number (La),

$$\text{We}=\frac{\rho_{f}{U_{I}}^{2}d_{p}}{\sigma_{f}}$$ (1)

and

$$\text{La}=\frac{\rho_{f}\sigma_{f}d_{p}}{{\mu_{f}}^{2}},$$ (2)

where *ρ*_*f*_, *μ*_*f*_, and *σ*_*f*_ are the fluid density, viscosity, and surface tension, respectively. The droplet Weber number (1) describes the ratio between the kinetic energy and the surface energy of a droplet moving at an incident normal impact velocity *U*_*I*_. The droplet Laplace number (2) represents the ratio of surface tension to the momentum-transport of a droplet moving in a fluid medium.

We extended the type of interaction mode to account for droplet-permeable-wall local interactions, as illustrated in Fig. 5. The interaction mode is a function of critical values of *We*, *La*, as well as that of a critical droplet diameter ${d_{p}}_{c}$ and the splash kinetic energy *E*_*KS*_ (see Ref. 46 for more details). The critical diameter is related to the filter microstructure, e.g., roughness, porosity, fibrous microstructure, and fibers orientation, i.e., it depends on the filter critical effective pore size and the initial size distribution of the saliva droplets. For example, Leonas and Jones^47^ reported a maximum pore size varying between 27.19 *µ*m and 146.6 *µ*m for six different types of face masks. Thus, any choice of ${d_{p}}_{c}$ between 27.19 *µ*m and 146.6 *µ*m, which fits the initial mask efficiency value correctly and lies in the initial size distribution, is considered appropriate. We present the different regime transition states of the local interaction modes (Fig. 5) for a saliva droplet impacting a fibrous porous filter in Table I. We developed a numerical model for the new interaction laws of Table I, thus taking into account the fibrous porous nature of the mask wall.

**TABLE I.** Regime transition state of the local interaction modes for a saliva droplet impacting a fibrous porous filter. *α* = 2630 and *β* = 0.183 were measured and reported for water droplets impacting on a dry surface.^45,46^ *α* = 1320 and *β* = 0.183 were reported for water droplets impacting on a wet surface;^45,46^ ${We_{c}}_{1}=0$ and ${d_{p}}_{c}=60\;\mu m$.

Interaction mode (see Fig. 5)	Condition	Critical number
Stick	$E_{KS}<{We_{c}}_{1}$	${We_{c}}_{1}$
Splash/Rebound	$\text{We}\leq {We_{c}}_{2}$	${We_{c}}_{2}=\alpha \;{\text{La}}^{-\beta }$
Penetrate	$d_{p}\leq {d_{p}}_{c}$	${d_{p}}_{c}$

The porosity of the mask *ε* was taken into account by imposing a resistance to bulk fluid flow, manifested by a pressure difference Δ*p* across the mask wall,

$$\frac{\Delta p}{d_{f}}=-D\;\mu_{f}\;U_{f}\;-\;0.5\;I\;\rho_{f}\;{\left|U_{f}\right|}^{2},$$ (3)

where *D* and *I* are the viscous (Darcy) and inertial coefficients, respectively. For a porous filter medium made of fiber,^48^ the coefficient *D* can be estimated as

$$D=\frac{64\;\xi^{1.5}\left(1+56\;\xi^{3}\right)}{{{d_{p}}_{c}}^{2}},$$ (4)

where *ξ* is the packing density of the fibrous porous material. We used *ξ* = 0.15 based on averages^49^ for fibrous layers of face masks.

According to the study of Jaksic and Jaksic,^50,51^ we estimate the inertial coefficient *I* as

$$I=\frac{1}{1.28^{2}}\;\frac{1}{0.5\;d_{f}},$$ (5)

where *d*_*f*_ is the face mask filter thickness taken as 2 mm. *d*_*f*_ depends on the manufacturing process, the folding techniques of the fibrous layers inside the mask, and the mask usage.

## III. RESULTS AND DISCUSSION

### A. Cough dynamics

**FIG. 6.**
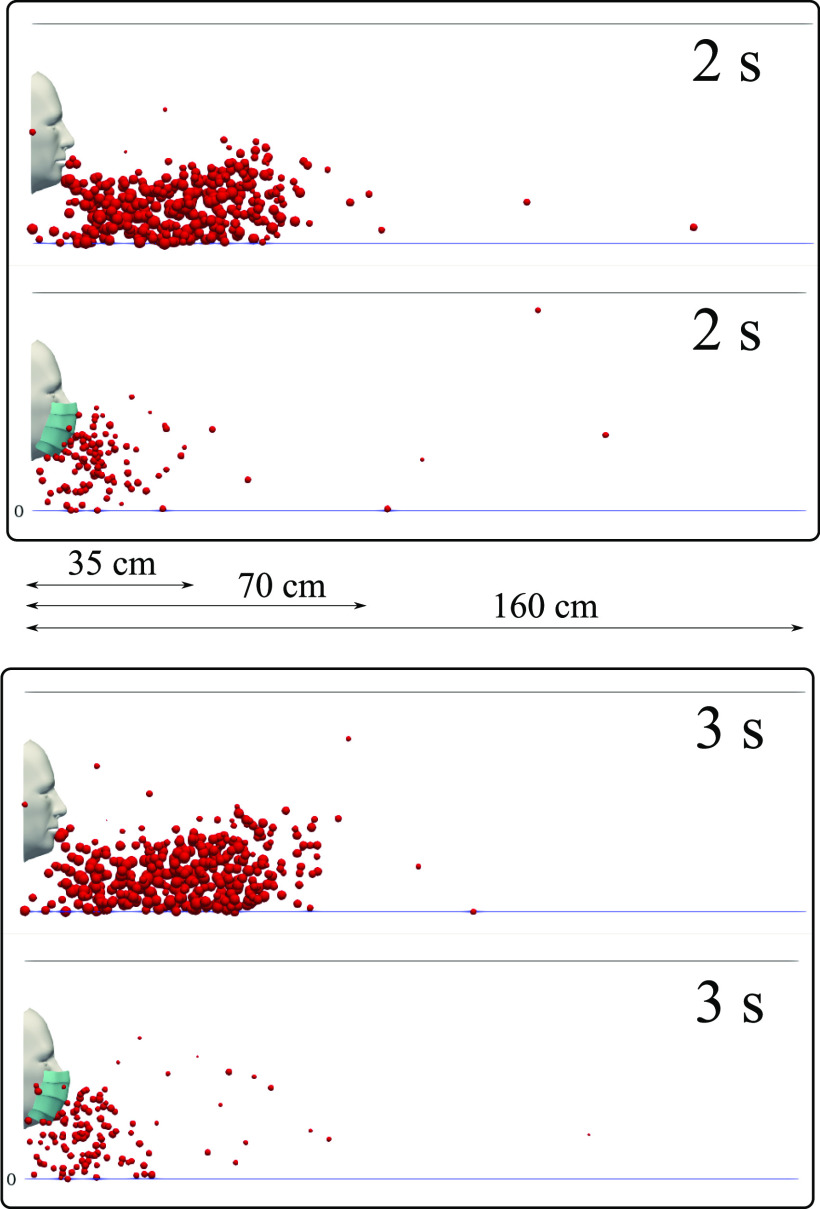
A subject coughing in a cyclic incident. A qualitative examination of airborne droplet transmission with and without wearing a surgical mask. The top and bottom figures show the results at 2 s and 3 s, respectively. Wearing a surgical mask that exhibits an initial efficiency of ∼91%. This cannot prevent the transport of the saliva droplets away from the subject. Many droplets penetrate the mask shield and some saliva droplet disease-carrier particles can travel more than 1.2 m. For visualization, the droplets were scaled by a factor of 600 compared to their actual size. The environmental conditions are zero wind speed, ambient temperature 20 °C, pressure 1 atm, and relative humidity 50%. The mouth temperature is 34 °C and the face skin temperature is 32 °C.

**FIG. 7.**
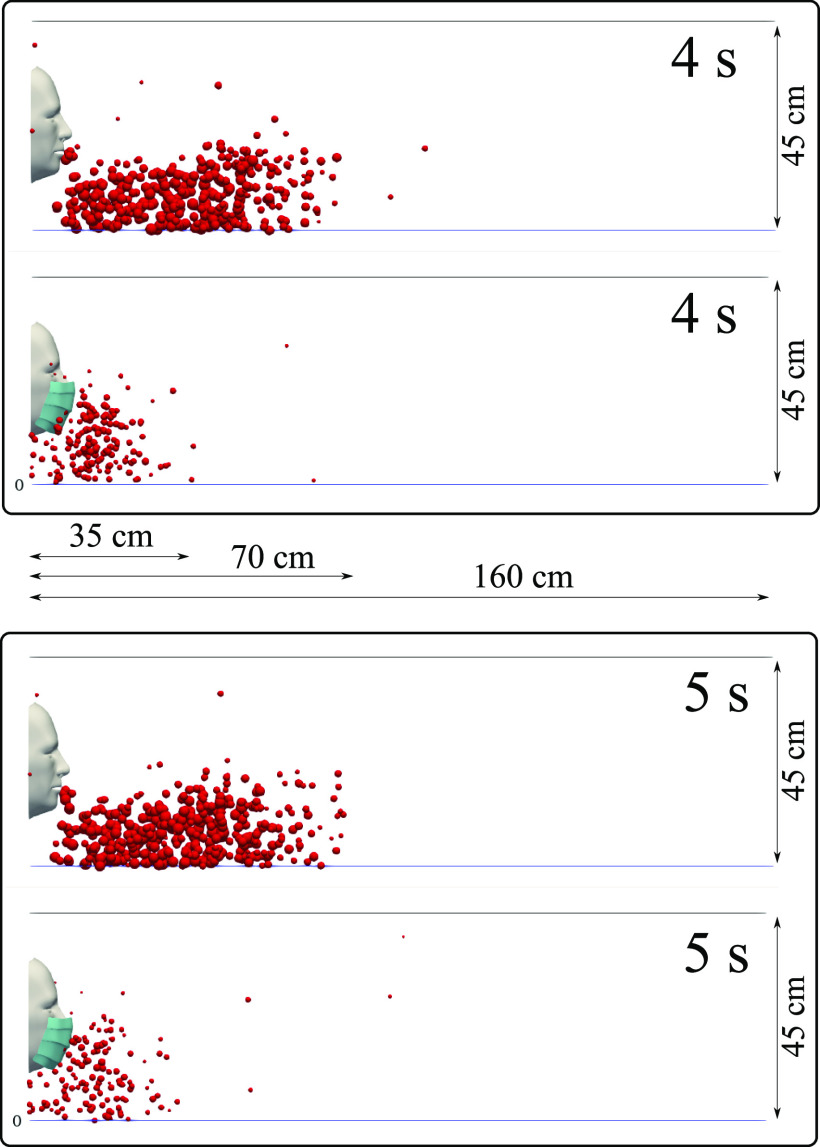
A subject coughing in a cyclic incident. A qualitative examination of airborne droplet transmission with and without wearing a surgical mask. The top and bottom figures show the results at 4 s and 5 s, respectively. Wearing a surgical mask that exhibits initial efficiency of ∼91%. This cannot prevent the transport of the saliva droplets away from the subject. Many droplets penetrate the mask shield and some saliva droplet disease-carrier particles can travel more than 1.2 m. For visualization, the droplets were scaled by a factor of 600 compared to their actual size. The environmental conditions are zero wind speed, ambient temperature 20 °C, pressure 1 atm, and relative humidity 50%. The mouth temperature is 34 °C, and the face skin temperature is 32 ° C.

**FIG. 8.**
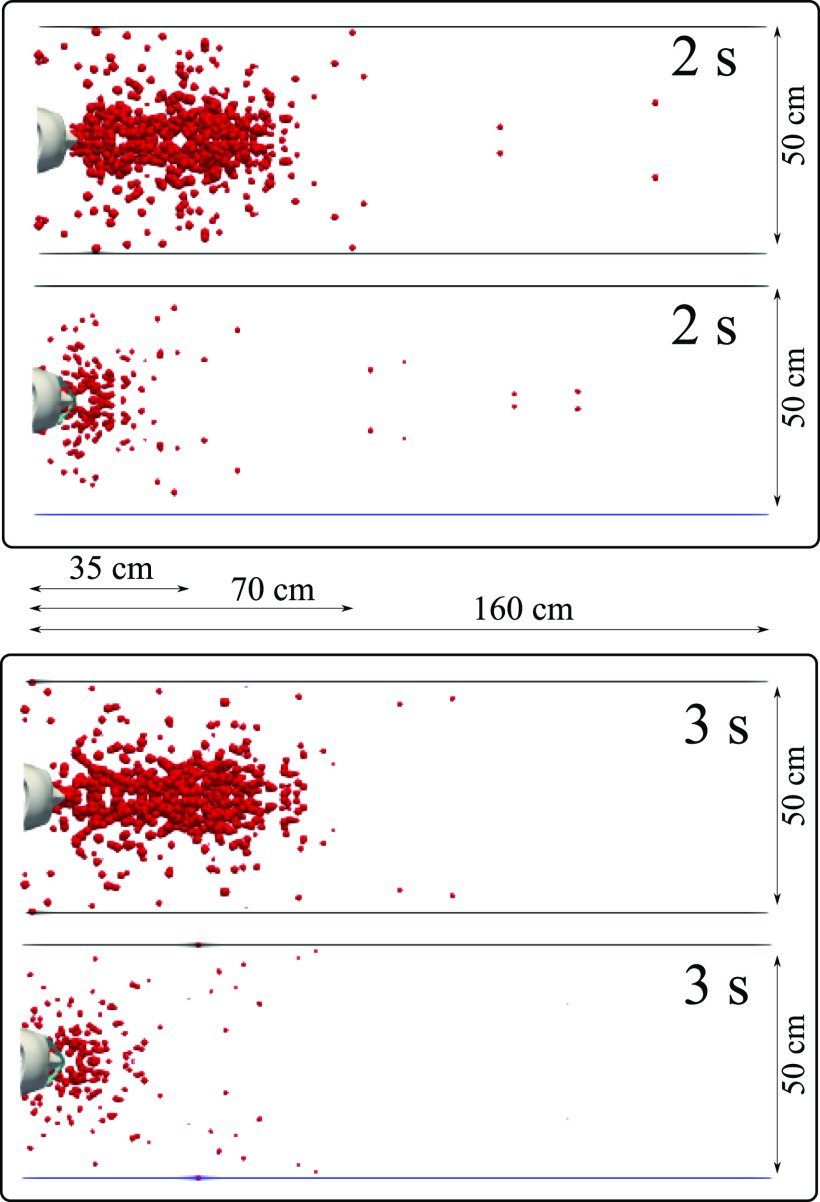
A subject coughing in a cyclic incident. Top view of a qualitative examination of airborne droplet transmission with and without wearing a surgical mask. The top and bottom figures show the results at 2 s and 3 s, respectively. We consider a surgical mask that exhibits initial efficiency of ∼91%. The cover does not prevent the transport of the saliva droplets entirely away from the subject. Many droplets penetrate the mask shield, and some saliva droplet disease-carrier particles can travel more than 1.2 m. For visualization, the droplets were scaled by a factor of 600 compared to their actual size. The environmental conditions are zero wind speed, ambient temperature 20 °C, pressure 1 atm, and relative humidity 50%. The mouth temperature is 34 °C, and the face skin temperature is 32 °C.

**FIG. 9.**
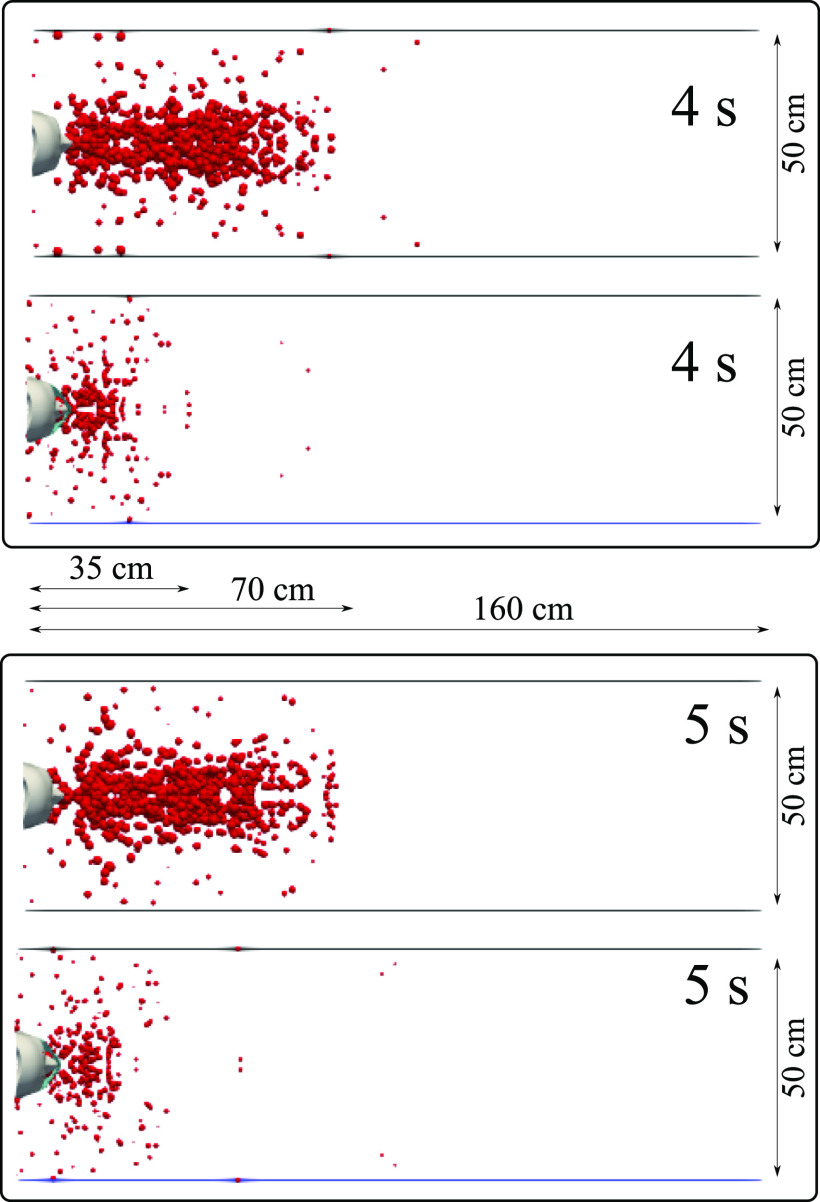
A subject coughing in a cyclic incident. Top view of airborne droplet transmission with and without wearing a surgical mask. The top and bottom figures show the results at 4 s and 5 s, respectively. We consider a surgical mask that exhibits an initial efficiency of ∼91%. The cover does not prevent the transport of the saliva droplets entirely away from the subject. Many droplets penetrate the mask shield and some saliva droplet disease-carrier particles can travel more than 1.2 m. For visualization, the droplets were scaled by a factor of 600 compared to their actual size. The environmental conditions are zero wind speed, ambient temperature 20 °C, pressure 1 atm, and relative humidity 50%. The mouth temperature is 34 °C, and the face skin temperature is 32 °C.

We carried out simulations for a subject with and without a mask and compared the airborne droplet transmission qualitatively at different time instants (Figs. 6–9). Wearing a mask close enough to the face significantly reduces the droplet cloud. However, some droplets still continue traveling to considerable distance, even further than 1 m (see at 2 s and 3 s in Fig. 6). Wearing a mask also reduces the lateral dispersion, but it does not eliminate it (Figs. 8 and 9). As we will show in Sec. III C, the nominal efficiency of the present mask is ∼91%. The simulations reveal that despite the high (nominal) efficiency, there is a considerable amount of droplets transported downstream of the subject. We will explain in detail what underpins the above behavior in Secs. III B and III C. A direct quantitative comparison of the results of Figs. 6–9 with the results of Dbouk and Drikakis^3^ cannot be made because, in the present study, we model a cyclic coughing incident. We explain the cough dynamics as follows:

1. Cyclic coughing induces more droplet-to-droplet and fluid-to-droplet interactions.
2. The expelled droplet jet over cycles pushes the droplets in front of the subject further downstream, thus increasing the distance to ∼70 cm (without mask) after 2 s, with some droplets still traveling beyond.
3. The droplet cloud also acts as a cushion for the expelled droplet jet over cycles, thus increasing the droplet residence time in front of the mouth.

**FIG. 10.**
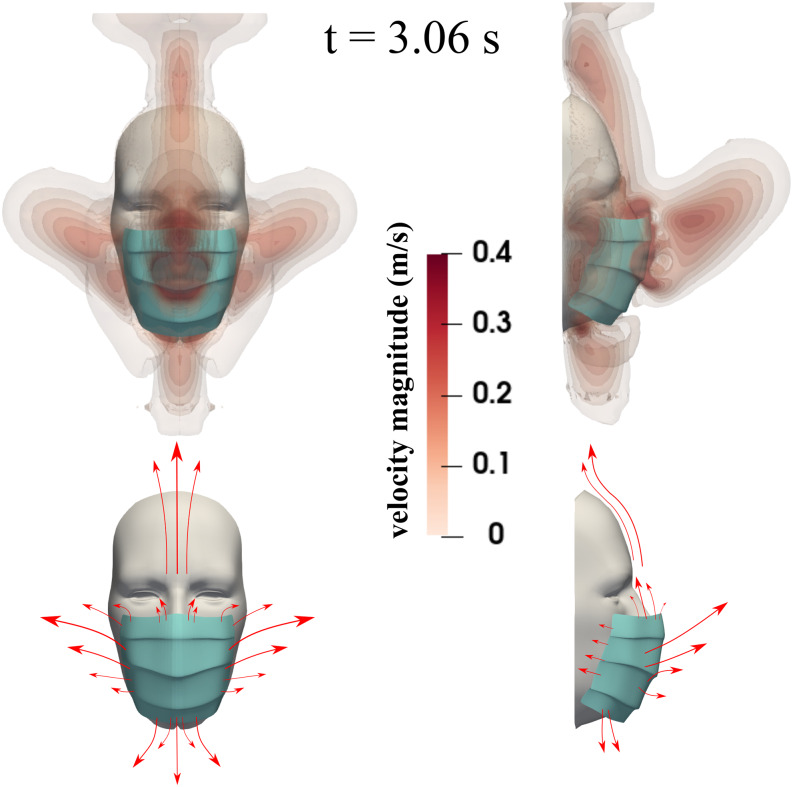
A subject coughing while wearing a surgical mask. The top figures show the velocity magnitude contours at t = 3.06 s. The bottom figures show a schematic of the flow dynamics.

**FIG. 11.**
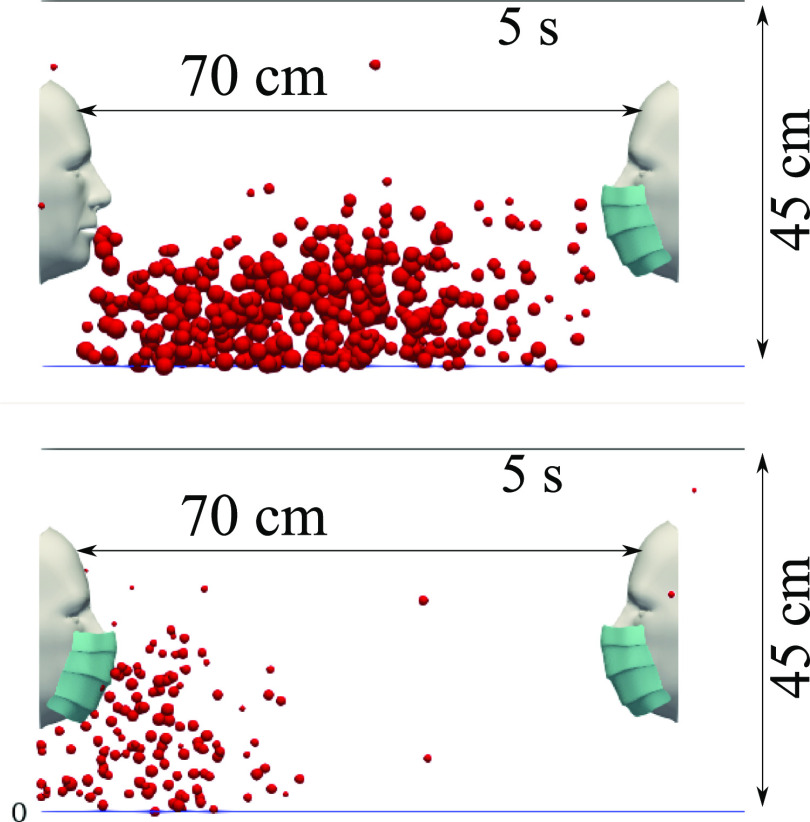
Mask wearer: subjects wearing a mask will reduce the respiratory droplet transmission while (partially) shielding themselves from other subjects experiencing a coughing incident. We show the results at 5 s simulation time for a surgical mask exhibiting an initial efficiency of ∼91%. The environmental conditions are zero wind speed, ambient temperature 20 °C, pressure 1 atm, and relative humidity 50%. The mouth temperature is 34 °C and the face skin temperature is 32 °C.

We show the flow dynamics around the face mask during the coughing incident at *t* = 3.06 s in Fig. 10. We have chosen a specific velocity range for visualization purposes, i.e., up to 0.4 m/s instead of the actual maximum velocity 5 m/s. At the top, we show the velocity magnitude contours for front and side views. The bottom figures are a schematic representation of how the flow escapes from the mask. There is considerable flow dynamics around the cover facilitated by a pressure differential between the space engulfed by the mask and the surrounding environment. The fluid escapes from all openings and carries with it the respiratory droplets we showed in Figs. 6 and 7. The droplet leakage due to the flow dynamics around the mask contributes to the cumulative increase of droplets during cough cycles, which we will also discuss quantitatively later on. For a mask filter with an initial efficiency of ∼91% and environmental conditions of zero wind speed, subjects wearing a mask will not only reduce the respiratory droplet transmission but also (partially) shield themselves from the droplet jet expelled from other subjects in their proximity. The results of Fig. 11 correspond to a 5 s simulation time.

**FIG. 12.**
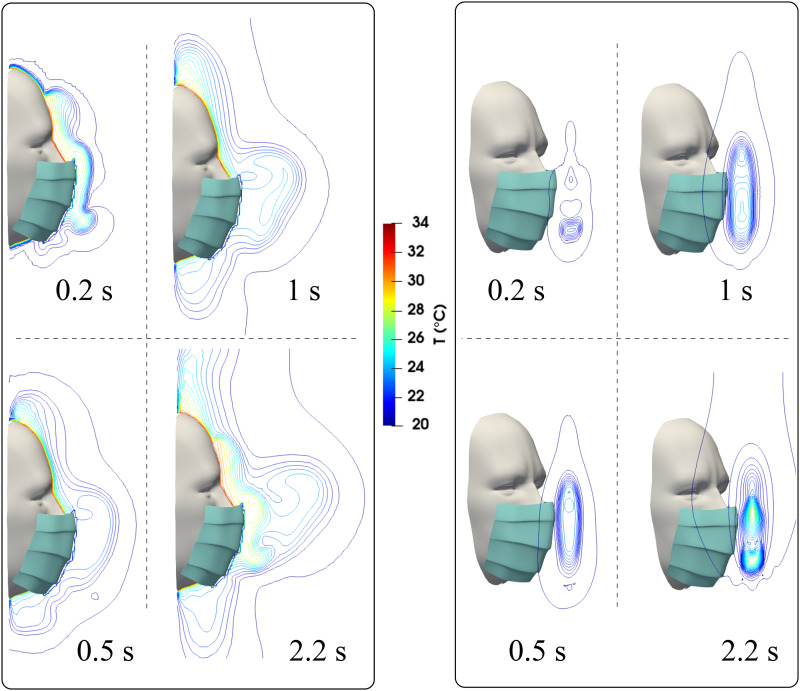
A subject coughing while wearing a surgical mask. The figures show the temperature contours at different times and side and perspective views.

In addition to the flow dynamics, buoyancy also contributes to the overall complexity. The different temperatures between the mouth at 34 °C, skin temperature at 32 °C, and the ambient air at 20 °C generate thermal gradients around the mask (Fig. 12). The higher temperature contour lines near the face skin manifest the above behavior. Buoyancy will facilitate the droplet leakage, particularly from the top of the mask. At *t* = 2.2 s, a thermal plume results in a hot spot circulation at the front of the mask (see the perspective view at *t* = 2.2 s). This effect is due to the accumulation of hot air from both mouth and nose ejections and droplet collisions downstream of the mask during cough cycles.

### B. Airborne droplets transmission

The droplet sizes change and fluctuate continuously during cough cycles as a result of several interactions with the mask and face, evaporation, breakup, and coalescence phenomena. Therefore, we have implemented the Sauter Mean Diameter (SMD), a classical averaging technique,^52^ to better quantify the evolving saliva droplet size. The SMD, also known as D32, is widely used in spray characterization. It represents the diameter of a droplet whose ratio of volume to surface area is equal to that of the total volume of all the droplets to their entire surface area. We adopted D32 because some droplets leave the domain and can alter the actual droplet size distribution. Thus, considering the finite domain size, D32 is a representative measure of droplet evolution because at each time instant, it is applied to all the droplets that are inside the domain.

**FIG. 13.**
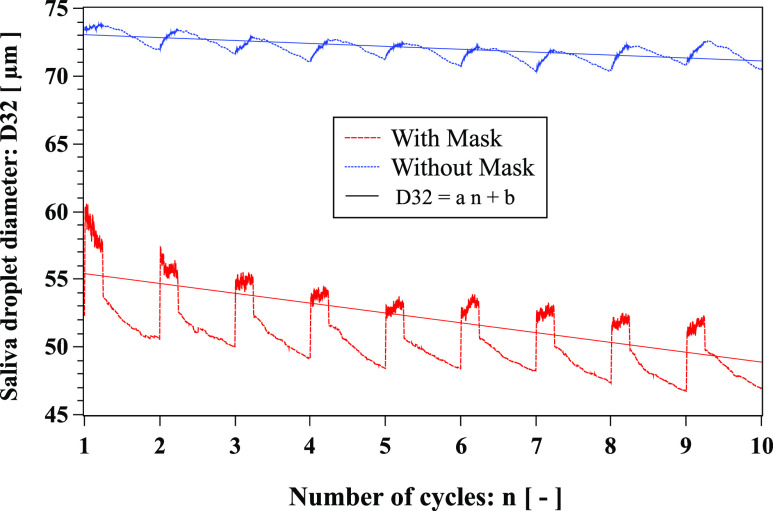
Evolution of respiratory droplet Sauter Mean diameter (SMD), known as *D*32, over ten cough cycles. Linear fit: with mask (*a* = −0.725, *b* ≈ 56.14) and without mask (*a* = −0.215, *b* ≈ 73.27). The environmental conditions are zero wind speed, ambient temperature 20 °C, pressure 1 atm, and relative humidity 50%. The mouth temperature is 34 °C and the temperature of the face skin is 32 °C.

D32 is higher for the case without a mask compared to the scenario with Fig. 13. Without a mask, D32 decreases with the number of cycles with a negative slope rate of −0.215. With a mask, D32 also decreases with the number of cycles but with a negative slope rate of −0.725. The highest decreasing rate for the mask scenario is due to the larger droplets sticking to the mask fibrous layers. Smaller droplets penetrate the mask and rebound or splash also occur, leading to droplet leakage from the cover (Figs. 5–9).

**FIG. 14.**
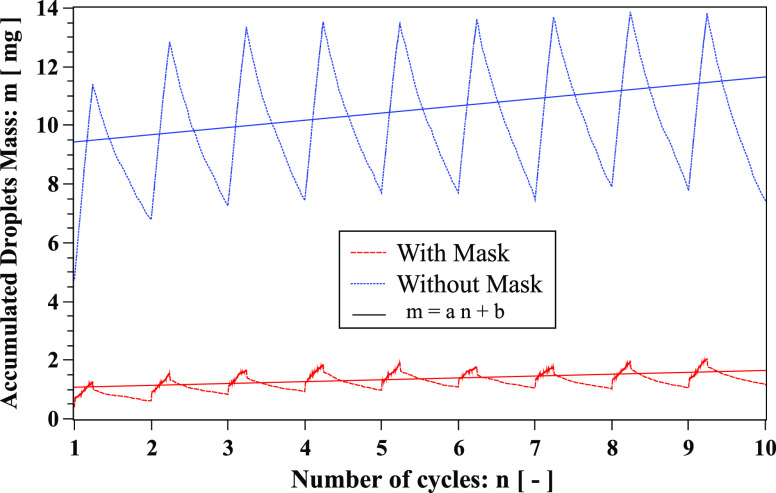
Accumulated mass of respiratory droplets over ten cough cycles. Linear fit: with mask (*a* = 0.063, *b* ≈ 1.01) and without mask (*a* = 0.24, *b* ≈ 9.19).

**FIG. 15.**
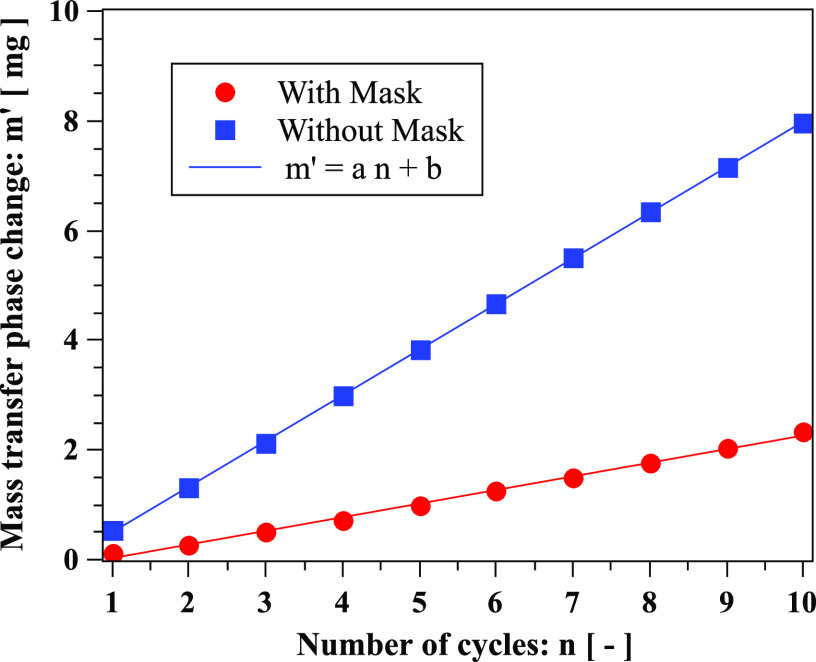
Mass transfer due to phase change (evaporation) of respiratory droplets over ten cough cycles. Linear fit: with mask (*a* = 0.248, *b* ≈ 0.22) and without mask (*a* = 0.832, *b* ≈ 0.33).

**FIG. 16.**
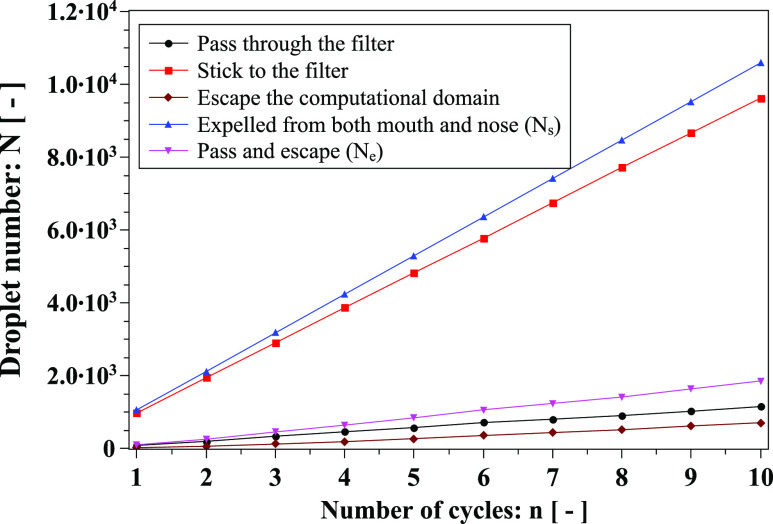
Analysis of different droplet types over cough cycles. *N*_*s*_ and *N*_*e*_ appear in Eqs. (6) and (7).

**FIG. 17.**
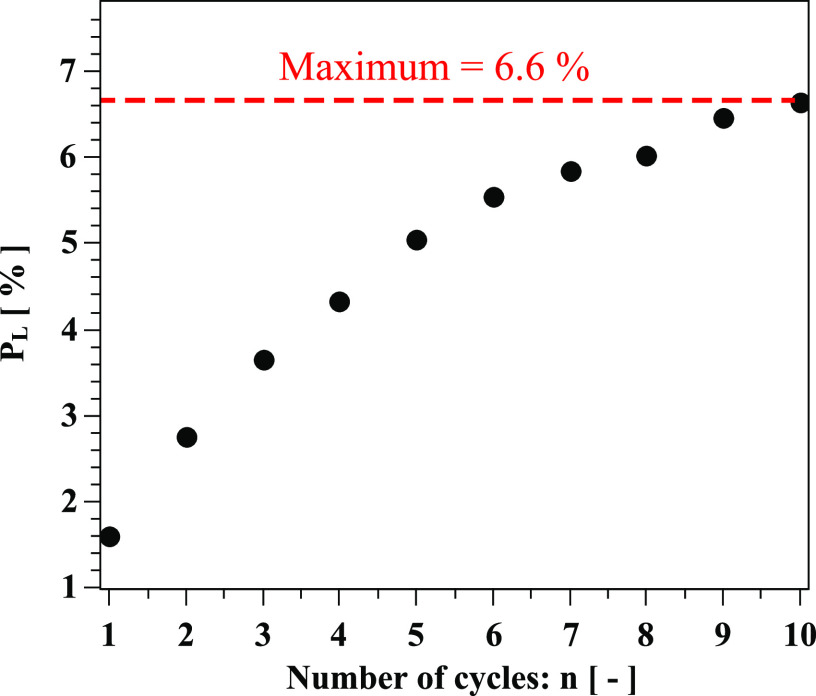
Uncertainty quantification: percentage of droplets leaving the computational domain during cough cycles regarding the total droplets expelled from both the mouth and nose.

We found that the accumulated droplet mass during cough cycles is reduced more when wearing a face mask (Fig. 14). The reduction is due to a combination of mask filtration, droplets leaving the computational domain, and droplet evaporation. To quantify the droplet evaporation, we calculate the mass transfer through a phase change, *m*′ (Fig. 15). The computational domain is finite; hence, some droplets escape the computational box. The accumulated mass comprises respiratory droplets inside the computational domain. It includes the total droplets expelled from the mouth and nose, excluding those sticking onto the mask and leaving the computational box or evaporating (phase change). We present the analysis of different droplet types in Fig. 16. The mask captures a significant number of droplets compared to those passing through. The slope difference between the blue (“expelled from both mouth and nose”) and red (“stick to filter”) lines represent the mask efficiency reduction over cycles. Furthermore, we quantify the uncertainty regarding droplets leaving the computational domain over ten cycles by plotting their maximum percentage, P_L_, compared to the total number expelled from both mouth and nose (Fig. 17). P_L_ exceeds 5% after the fifth cycle and does not exceed 6.6% at the tenth cycle.

### C. Penetration criterion

**FIG. 18.**
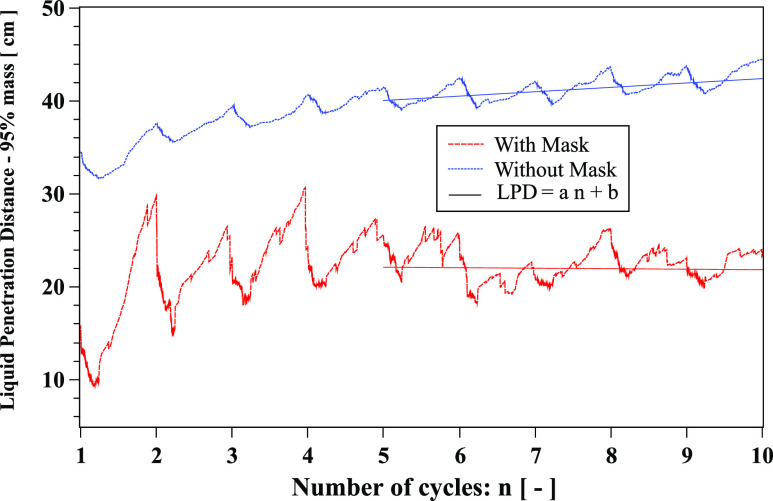
Liquid Penetration Distance (LPD) evolution over ten cough cycles. Linear fit: with mask (*a* = −0.05, *b* ≈ 22.38) and without mask (*a* = 0.47, *b* ≈ 37). The environment is at ambient temperature, pressure, and relative humidity of 20 °C, 1 atm, and 50%, respectively. The mouth temperature is 34 °C and the face skin is 32 °C. The results are plotted from the end of the first cycle.

The Liquid Penetration Distance (LPD) is an important parameter that describes the maximum distance traveled by a saliva liquid droplet made of 95% of its initial mass. Figure 18 shows that LPD reduces from about 42 cm to 22.38 cm after the tenth cycle when wearing a face mask. This value is of the same order of magnitude compared to the experimental data of Hui *et al.*^4^

**FIG. 19.**
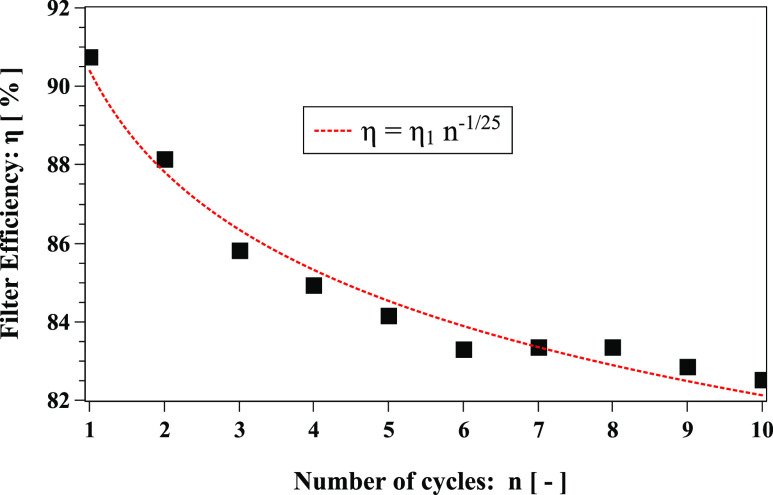
Filter efficiency over ten cough cycles. *η*_1_ = *η*(*n* = 1) = 90.4% (*R*^2^ = 0.97).

For the first time, we introduce an effective dynamic filter efficiency *η* that is a function of the number of cycles *n* of a coughing incident. It reflects the total number of saliva droplets escaping the face mask,

$$\eta \;=\;1-PR\left(n\right)\;=\;\eta_{1}\;n^{\gamma },$$ (6)

where *PR* denotes the mask penetration ratio

$$PR\left(n\right)=\frac{N_{e}\left(n\right)}{N_{s}\left(n\right)},$$ (7)

where *N*_*s*_(*n*) and *N*_*e*_(*n*) are the number of droplets at the start and at the end of the *n*th cycle, respectively; *η*_1_, in (6), denotes the initial mask efficiency (≈91%) that depends purely on the filter material properties applied in the mask layers. The exponent *γ* is a coefficient that describes the transient effect of the fluid flow and droplet dynamics altering the mask efficiency. For the conditions considered in this study, we obtained *γ* = −1/25 (Fig. 19). The negative sign of *γ* denotes a degradation of the mask efficiency with time. To our knowledge, mask manufacturers provide only *η*_1_, which cannot describe degradation mask efficiency due to dynamic coughing incidents (Fig. 19).

## IV. CONCLUSIONS AND RECOMMENDATIONS

We computationally investigated the flow physics of respiratory droplets arising from coughing around and through a face mask. We considered a mask consisting of air-permeable filtering material made of porous fibrous layers. The fluid flow and cough dynamics significantly influence the droplet transmission and, in turn, the overall mask efficiency:

1. Without wearing a mask, droplets travel to about 70 cm.
2. Wearing a mask, the bulk of droplets will travel about half the distance.
3. However, in both cases, there are still isolated droplets transmitted beyond 70 cm.
4. Mask efficiency is dynamic (not constant). It is reduced during cough cycles. The fluid dynamics and the interactions between the droplets, the filter, and the face influence mask efficiency. We show that after ten cough cycles, efficiency can drop ∼8%. The above is a conservative prediction considering that we model a mild cough incident and ten cough cycles. We should expect more significant efficiency reduction for severe coughing events, as well as when wearing a mask for a longer period.
5. The dosage and time of exposure to a virus affecting a human are not known and will vary across subjects. We examined 10% and 32% of droplets, which are smaller than their corresponding initial size, and found that they reduce in number during cough cycles when wearing a mask.
6. The diameter of the transmitted droplets is larger across cough cycles when no mask is worn.
7. The accumulation of droplets in the surrounding environment increases as the cough continues and is more significant without a mask.
8. With a mask, droplet penetration approximately reaches a mean value. Without a mask, the rate of the droplet penetration increases with cough cycles and tends to decrease after several periods.
9. The mask to face fitting is important. Even in the case of a tight fitting scenario, if there exist some small openings, this can lead to additional leakage of droplets around the mask, which cannot be ignored. It contributes to an additional reduction in the mask efficiency with respect to efficiency reduction induced by the cyclic behavior of the coughing incident.
10. By wearing a mask, it will also provide greater protection to the wearer as it blocks the droplets expelled from another subject and further decelerates the incoming jet.
11. The complex droplet interactions and fluid physics lead to interesting phenomena such as hot spots downstream of the mask and flow recirculation associated with buoyancy.

According to the results of this study, we make the following recommendations:

1. Although masks will reduce droplet transmission, we should not ignore that several droplets will be transmitted away from the mask. The use of a mask will not provide complete prevention from airborne droplet transmission. The above is particularly important for both indoor and outdoor environments. As Dbouk and Drikakis^3^ showed, respiratory droplets can be transmitted to several meters away from the subject due to wind conditions. Therefore, social distancing remains essential when facing an evolving pandemic.
2. The above recommendation implies that we can protect healthcare workers only if we equip them with a complete PPE, e.g., a helmet with a built-in air filter, a face shield together with a disposable suit over the whole ensemble, and a double set of gloves.
3. The manufacturers and regulatory authorities should consider new criteria for assessing mask performance to account for the flow physics and cough dynamics. We provided a simple criterion that takes into account efficiency reduction during a cyclic coughing incident.

Further research is required to advance the understanding of the following:

1. Droplets breakup and coalescence phenomena that induce a liquid film barrier on the fibrous porous surface of the face mask, e.g., at the pore microstructure scale.
2. Cough dynamics across subjects that experience different medical conditions.
3. Saliva droplet composition effects on cough dynamics and droplet transmission.
4. The effects of a high-filter efficiency offered by more advanced mask designs relative to breathing comfort.

## SUPPLEMENTARY MATERIAL

See the supplementary material for the respiratory droplets and face masks.

## DATA AVAILABILITY

The data that support the findings of this study are available from the corresponding author upon reasonable request.
